# Significant Decrease in Pertactin-Deficient *Bordetella pertussis* Isolates, Japan

**DOI:** 10.3201/eid2304.161575

**Published:** 2017-04

**Authors:** Yukihiro Hiramatsu, Yusuke Miyaji, Nao Otsuka, Yoshichika Arakawa, Keigo Shibayama, Kazunari Kamachi

**Affiliations:** National Institute of Infectious Diseases, Tokyo, Japan (Y. Hiramatsu, Y. Miyaji, N. Otsuka, K. Shibayama, K. Kamachi);; St. Marianna University School of Medicine, Kawasaki, Japan (Y. Miyaji);; Nagoya University Graduate School of Medicine, Nagoya, Japan (Y. Arakawa)

**Keywords:** *Bordetella pertussis*, pertactin, genotype, multilocus variable-number tandem-repeat analysis, bacteria, pertussis, whooping cough, respiratory infections, Japan

## Abstract

Prevalence of pertactin-lacking *Bordetella pertussis* isolates has been observed worldwide. In Japan, however, we found that the frequency of pertactin-deficient isolates in 2014–2016 (8%) was significantly lower than the frequency in 2005–2007 (41%), 2008–2010 (35%), and 2011–2013 (25%). This reduction was closely associated with changes in genotypes.

*Bordetella pertussis*, a highly communicable, gram-negative coccobacillus, is the etiologic agent of pertussis (whooping cough), an acute respiratory infection that leads to severe illness in children. Vaccination is the most effective method for preventing and controlling pertussis. In Japan, acellular pertussis vaccines (ACVs) were introduced in 1981. Pertussis toxin and filamentous hemagglutinin derived from *B. pertussis* are the major antigens in ACVs in Japan, and certain ACVs also contain pertactin and fimbriae (*1*). Pertactin is believed to play a role in adherence to human epithelial cells (*2*); however, *B. pertussis* isolates that lack pertactin production have been identified in several countries where ACVs have been introduced (*3*–*7*). In Japan, pertactin-deficient isolates have increased significantly since the early 2000s, resulting in a high prevalence of these isolates (*5*,*8*). Recent studies have demonstrated that pertactin-deficient strains could colonize the respiratory tract more effectively than pertactin-producing strains in ACV-vaccinated mice (*9*,*10*). Supporting these results, an epidemiologic study suggested that ACV-vaccinated persons are more susceptible to pertactin-deficient strains than to pertactin-producing strains (*11*). These reports imply that pertactin-deficient strains have increased fitness in humans who have been vaccinated with ACVs and that their expansion may reduce the effectiveness of ACVs. We assessed trends in the frequency of pertactin-deficient isolates in Japan and further investigated their genotypes using multilocus variable-number tandem-repeat analysis (MLVA).

## The Study

We studied 232 *B. pertussis* clinical isolates collected from January 2005 through June 2016 in Japan. All isolates were derived from epidemiologically unrelated cases of pertussis. Pertactin production and MLVA types (MTs) of 111 isolates collected during 2005–2012 were previously determined by immunoblotting and MLVA typing, respectively (*5*,*8*). For our study, we extended these analyses to additional isolates collected during 2008–2016 (n = 121).

We determined the temporal trend in the frequency of pertactin-deficient isolates by 3-year periods (Figure, panel A). Percentages were 41% in 2005–2007 (n = 39 isolates), 35% in 2008–2010 (n = 43), 25% in 2011–2013 (n = 97), and 8% in 2014–2016 (n = 53). A significant decrease in the frequency of pertactin-deficient isolates was observed from 2005–2007 to 2014–2016 (p<0.05 by Fisher exact test). 

**Figure:**
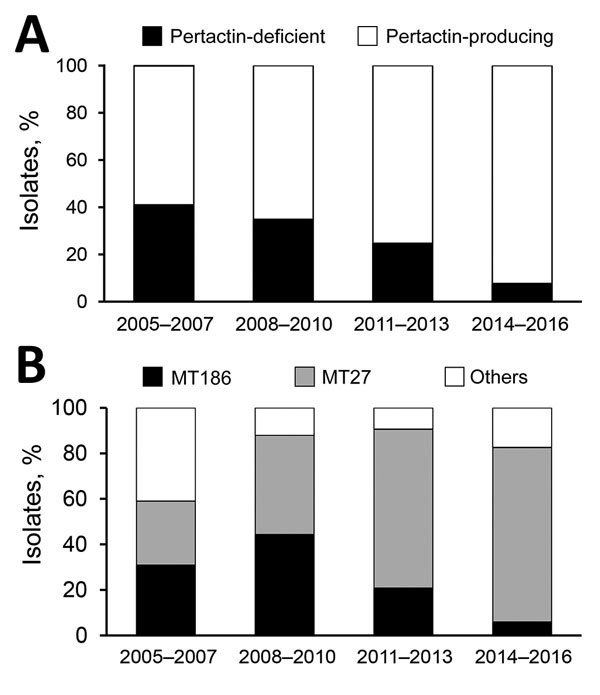
Temporal trends in the frequency of pertactin-deficient isolates and changes in multi-locus variable-number tandem repeat analysis types (MTs) in the *Bordetella pertussis* population in Japan. Pertactin production (A) and MTs (B) were analyzed for 232 *B. pertussis* isolates collected from January 2005 through June 2016. The frequencies of pertactin-deficient isolates and 2 major MTs (MT27 and MT186) are shown by time period. For convenience, minor MTs (MT194, MT224–226, and MT314–316) are included as “others.”

Among the 232 *B. pertussis* isolates, 25 MTs were identified; MT27 and MT186 isolates were predominant, and other MT isolates were found at low frequencies (Technical Appendix Table 1). The frequency of MT27 isolates increased significantly over time (Figure 1, panel B): 28% in 2005–2007, 44% in 2008–2010, 70% in 2011–2013, and 77% in 2014–2016. In contrast, the frequency of MT186 isolates decreased: 31% in 2005–2007, 44% in 2008–2010, 21% in 2011–2013, and 6% in 2014–2016. We also observed a substitution of the major genotype in the *B. pertussis* population from MT186 to MT27.

Of 59 pertactin-deficient *B. pertussis* isolates collected during 2005–2016, 45 (76.3%) were MT186 isolates, whereas 2 (3.4%) represented MT27 and 12 (20.3%) other MT isolates (MT194, MT224–226, MT314–316) (Table). Notably, 45 (83.3%) of 54 MT186 isolates were pertactin-deficient, whereas only 2 (1.4%) of 139 MT27 isolates were pertactin-deficient. This finding indicates that pertactin-deficient isolates predominate among the MT186 strain but are rare among the MT27 strain.

**Table:** Molecular mechanisms of loss of pertactin production in 59 pertactin-deficient *Bordetella pertussis* isolates, Japan, 2005–2016*

Reason for loss of pertactin	No. (%) MTs		
MT27	MT186	Others†
ΔSS	0	43 (72.9)	11 (18.6)
1598–1599::IS481	0	2 (3.4)	1 (1.7)
245–246::IS481	1 (1.7)	0	0
Transcriptional downregulation	1 (1.7)	0	0
Total	2 (3.4)	45 (76.3)	12 (20.3)

*MT, types determined by using multilocus variable-number tandem-repeat analysis. †For convenience, minor MTs (MT194, MT224–226, and MT314–316) are included in this category.

We previously showed that pertactin-deficient isolates in Japan were generated by 2 different mutations: an 84-bp deletion of the *prn* gene signal sequence (ΔSS) and an IS*481* insertion at nucleotide position 1598 in *prn* (1598–1599::IS*481*) (*5*). Thus, to confirm the molecular basis for the loss of pertactin production, pertactin-deficient isolates (n = 26) that were newly identified in this study underwent PCR screening with 2 primer sets (Technical Appendix Table 2). We summarized the molecular mechanisms of loss of pertactin production in 59 pertactin-deficient isolates (Table); the ΔSS mutation was detected in 43 (72.9%) MT186 isolates and in 11 (18.6%) other MT isolates. In contrast, the 1598–1599::IS*481* mutation was detected in 2 (3.4%) MT186 isolates and 1 other MT isolate (1.7%, MT226). Two MT27 isolates (BP394 and BP533) do not have either of these mutations. Instead, BP533 isolates (1.7%) have an IS*481* insertion at nucleotide position 245 (245–246::IS*481*; GenBank accession no. KC445198), and BP394 (1.7%) isolates exhibit transcriptional down-regulation of *prn* gene expression (Technical Appendix Figure 1).

## Conclusions

The expansion of pertactin-deficient *B. pertussis* isolates has been reported worldwide (*3*–*8*). However, we observed a significant decrease in pertactin-deficient isolates within the *B. pertussis* population in Japan, caused by a genotypic replacement from the pertactin-deficient MT186 strain to the pertactin-producing MT27 strain.

The most likely explanation for the prevalence of pertactin-deficient strains is vaccine-driven strain evolution, because pertactin is a component of ACVs. No pertactin-deficient isolates have been detected in Denmark, where an ACV that does not contain pertactin is used, and pertactin-deficient strains exhibit a selective advantage against ACV-induced immunity (*7*,*9*–*11*). In Japan, 5 brands of the diphtheria–tetanus–acellular pertussis vaccine (DTaP) had been used to control pertussis for many years. These DTaPs had different formulations of components; only 3 contained the pertactin antigen (*1*). When the DTaP vaccine was replaced, however, 2 brands of combined DTaP–inactivated poliovirus (DTaP-IPV) vaccine that did not contain pertactin were introduced in November 2012. Therefore, most Japanese children <4 years of age do not have immunity to pertactin; consequently, the selective pressure for pertactin-deficient strains in the host environment has recently been reduced. This effect may be responsible for the recent decline in pertactin-deficient isolates and further supports the hypothesis that pertactin-deficient strains are selected on the basis of host immunity to pertactin. Notably, a new brand of DTaP-IPV vaccine containing pertactin was also introduced in December 2015 in Japan. If the hypothesis of vaccine-driven evolution is correct, pertactin-deficient isolates should increase again in Japan in the near future. Thus, continued surveillance of pertactin-deficient isolates is of particular value.

We demonstrated that genotypic replacement from MT186 to MT27 has taken place among recent *B. pertussis* isolates in Japan: MT27 is a triple-locus variant of MT186. MT186 strains carry the pertussis-toxin promoter allele *ptxP1*, whereas MT27 strains carry the allele *ptxP3* (*8*). *B. pertussis* strains carrying *ptxP3* (i.e., MT27) produce more of several virulence factors than do *ptxP1* (i.e., MT186) strains (*12*,*13*). The population of MT27 strains carrying *ptxP3* has increased worldwide (*14*,*15*), although a low frequency of *ptxP1* isolates was observed in Japan (*8*), suggesting that MT27 strains are associated with the recent pertussis resurgence. It is possible, therefore, that the genotypic replacement in the *B. pertussis* population may have resulted from the expansion of the more virulent *ptxP3* (i.e., MT27) strains. In addition, given that pertactin-deficient MT27 isolates are rare, this genotypic replacement may have contributed to the recent decrease in pertactin-deficient isolates in Japan.

In Japan, most pertactin-deficient isolates carry a deletion of the *prn* signal sequence (ΔSS), which has been found primarily in MT186 isolates carrying the *prn1* allele (Technical Appendix Table 1). In other countries, a common *prn* mutation includes an insertion of IS*481* into the *prn2* allele (*4*,*6*,*7*). In this study, we identified 2 pertactin-deficient MT27 isolates carrying the *prn2* allele, due to the IS*481* insertion (245–246::IS*481*) and the transcriptional down-regulation of the *prn* gene. These pertactin-deficient isolates were previously identified in Europe and the United States (*6*,*7*). One possible explanation for the appearance of pertactin-deficient MT27 isolates is that they were imported from other countries.

Technical Appendix.  Table showing characteristics of *Bordetella pertussis* isolates, Japan, 2005–2016, table showing primers used in the study of *B. pertussis* isolates, Japan, 2008–2016, and figure showing lack of *prn* transcript expression in pertactin-deficient *B. pertussis* isolate BP394.
