# Supplementary material for: Significant Decrease in Pertactin-Deficient Bordetella pertussis Isolates, Japan
Source: Emerg Infect Dis. 2017 Apr;23(4):699–701. doi: 10.3201/eid2304.161575 (PMC5367402; doi:10.3201/eid2304.161575)
Supplement: Supplementary file 1 — Technical Appendix. Table showing characteristics of Bordetella pertussis isolates, Japan, 2005–2016, table showing primers used in the study of B. pertussis isolates, Japan, 2008–2016, and figure showing lack of prn transcript expression in pertactin-deficient B. pertussis isolate BP394. [file 16-1575-Techapp-s1.pdf]

Article DOI: http://dx.doi.org/10.3201/eid2304.161575

Significant Decrease in Pertactin–Deficient *Bordetella pertussis* Isolates, Japan

Technical Appendix

Technical Appendix Table 1. Characteristics of *Bordetella pertussis* isolates, Japan, 2005–2016*

Isolate	Isolation year	Patient age	Vaccine status	Origin (district)	Pertactin production	*prn* gene	*prn* allele	MLVA type
BP285	2005	2 mo	0	Kanto	–	ΔSS	1	186
BP291	2005	1 y	3	Chubu	–	ΔSS	1	186
BP292	2005	1 mo	0	Chubu	–	ΔSS	1	226
BP293	2005	4 mo	1	Chubu	–	ΔSS	1	186
BP299	2005	5 mo	0	Kyusyu	–	ΔSS	1	186
BP302	2005	1 mo	0	Kinki	–	1598–1599::IS*481*	1	226
BP307	2006	2 mo	0	Chubu	–	1598–1599::IS*481*	1	186
BP310	2006	11 y	Unknown	Tohoku	–	ΔSS	1	194
BP313	2006	58 y	Unknown	Kanto	–	1598–1599::IS*481*	1	186
BP322	2006	1 mo	0	Kanto	–	ΔSS	1	186
BP323	2006	1 y	Unknown	Kanto	–	ΔSS	1	186
BP332	2006	35 y	Unknown	Kinki	–	ΔSS	1	194
BP333	2006	30 y	Unknown	Kinki	–	ΔSS	1	194
BP334	2006	0 y	0	Kinki	–	ΔSS	1	194
BP314	2007	3 mo	0	Kanto	–	ΔSS	1	186
BP318	2007	1 y	0	Kanto	–	ΔSS	1	186
BP466	2008	33 y	Unknown	Kanto	–	ΔSS	1	186
BP469	2008	8 y	Unknown	Kanto	–	ΔSS	1	186
BP346	2009	2 mo	0	Chubu	–	ΔSS	1	186
BP351	2009	4 mo	Unknown	Shikoku	–	ΔSS	1	186
BP458	2009	70 y	Unknown	Kanto	–	ΔSS	1	186
BP459	2009	5 mo	Unknown	Kanto	–	ΔSS	1	186
BP460	2009	6 mo	Unknown	Kanto	–	ΔSS	1	186
BP357	2010	12 y	Unknown	Kanto	–	ΔSS	1	186
BP358	2010	10 y	Unknown	Kanto	–	ΔSS	1	186
BP359	2010	Unknown	Unknown	Kanto	–	ΔSS	1	186
BP360	2010	Unknown	Unknown	Kanto	–	ΔSS	1	186
BP361	2010	10 y	Unknown	Kanto	–	ΔSS	1	186
BP363	2010	Unknown	Unknown	Kanto	–	ΔSS	1	186
BP365	2010	Unknown	Unknown	Kanto	–	ΔSS	1	186
BP366	2010	6 y	Unknown	Kanto	–	ΔSS	1	186
BP378	2011	21 y	Unknown	Kyusyu	–	ΔSS	1	186
BP390	2011	4 y	Unknown	Kinki	–	ΔSS	1	316
BP394	2011	5 mo	0	Kyusyu	–	*prn* (transcriptional down–regulation)	2	27
BP395	2011	6 mo	0	Kinki	–	ΔSS	1	186
BP397	2011	7 y	Unknown	Kinki	–	ΔSS	1	186
BP398	2011	4 mo	0	Kinki	–	ΔSS	1	225
BP400	2011	5 y	Unknown	Kinki	–	ΔSS	1	186
BP401	2011	9 y	4	Kinki	–	ΔSS	1	314
BP403	2011	2 mo	0	Kinki	–	ΔSS	1	315
BP417	2011	35 y	4	Kanto	–	ΔSS	1	186
BP437	2011	1 mo	0	Kinki	–	ΔSS	1	186
BP438	2011	2 mo	0	Kinki	–	ΔSS	1	186
BP451	2011	3 mo	0	Kinki	–	ΔSS	1	186
BP452	2011	7 mo	Unknown	Kinki	–	ΔSS	1	186
BP410	2012	1 mo	0	Tohoku	–	ΔSS	1	186
BP412	2012	26 y	Unknown	Shikoku	–	ΔSS	1	186

Isolate	Isolation year	Patient age	Vaccine status	Origin (district)	Pertactin production	*prn* gene	*prn* allele	MLVA type
BP416	2012	3 mo	0	Kanto	–	ΔSS	1	186
BP418	2012	5 y	4	Kanto	–	ΔSS	1	186
BP440	2012	10 y	4	Kinki	–	ΔSS	1	186
BP442	2012	2 y	0	Kinki	–	ΔSS	1	316
BP447	2012	28 y	Unknown	Kyusyu	–	ΔSS	1	186
BP478	2012	5 y	Unknown	Kinki	–	ΔSS	1	186
BP480	2013	2 mo	0	Kinki	–	ΔSS	1	224
BP481	2013	1 mo	0	Kinki	–	ΔSS	1	186
BP510	2015	1 mo	0	Kinki	–	ΔSS	1	186
BP533	2015	1 mo	0	Kinki	–	245-246::IS*481*	2	27
BP535	2015	3 mo	0	Kyusyu	–	ΔSS	1	186
BP550	2016	7 y	4	Kanto	–	ΔSS	1	186
BP283	2005	9 y	Unknown	Kanto	+	*prn*	2	27
BP284	2005	1 mo	0	Chubu	+	*prn*	1	186
BP289	2005	6 mo	0	Kinki	+	*prn*	2	27
BP290	2005	1 y	0	Chugoku	+	*prn*	2	27
BP294	2005	10 mo	1	Kyusyu	+	*prn*	2	27
BP296	2005	6 y	0	Kyusyu	+	*prn*	2	27
BP297	2005	3 mo	0	Chugoku	+	*prn*	2	22
BP298	2005	3 mo	0	Kinki	+	*prn*	2	69
BP300	2005	2 mo	0	Kyusyu	+	*prn*	2	26
BP301	2005	11 mo	0	Kyusyu	+	*prn*	2	27
BP303	2005	1 mo	0	Chugoku	+	*prn*	1	224
BP306	2006	10 y	4	Chubu	+	*prn*	2	69
BP311	2006	1 y	Unknown	Tohoku	+	*prn*	1	187
BP335	2006	0 y	Unknown	Kinki	+	*prn*	1	186
BP312	2007	4 mo	0	Kanto	+	*prn*	2	27
BP316	2007	53 y	Unknown	Tohoku	+	*prn*	1	234
BP317	2007	11 y	Unknown	Tohoku	+	*prn*	1	234
BP324	2007	65 y	Unknown	Kanto	+	*prn*	2	27
BP327	2007	10 y	Unknown	Shikoku	+	*prn*	1	229
BP330	2007	6 mo	0	Chubu	+	*prn*	2	27
BP336	2007	0 y	2	Kinki	+	*prn*	2	27
BP337	2007	43 y	Unknown	Kinki	+	*prn*	2	27
BP343	2007	0 y	Unknown	Kanto	+	*prn*	2	95
BP331	2008	1 y	0	Chubu	+	*prn*	2	27
BP338	2008	11 y	2	Kinki	+	*prn*	2	27
BP339	2008	8 y	Unknown	Kinki	+	*prn*	2	27
BP340	2008	29 y	Unknown	Kinki	+	*prn*	2	27
BP341	2008	6 y	Unknown	Kinki	+	*prn*	1	187
BP342	2008	0 y	Unknown	Kinki	+	*prn*	2	27
BP344	2008	5 y	Unknown	Chubu	+	*prn*	2	27
BP345	2008	4 y	Unknown	Chubu	+	*prn*	2	27
BP464	2008	3 mo	0	Kanto	+	*prn*	2	27
BP465	2008	27 y	Unknown	Kanto	+	*prn*	2	27
BP467	2008	8 mo	Unknown	Kanto	+	*prn*	2	27
BP347	2009	3 y	0	Shikoku	+	*prn*	9	27
BP348	2009	41 y	Unknown	Chubu	+	*prn*	2	27
BP349	2009	2 mo	0	Kyusyu	+	*prn*	1	186
BP350	2009	13 y	4	Chubu	+	*prn*	1	186
BP352	2009	1 mo	0	Shikoku	+	*prn*	1	34
BP353	2009	12 y	Unknown	Kyusyu	+	*prn*	2	22
BP354	2009	1 mo	0	Kyusyu	+	*prn*	1	311
BP355	2009	6 mo	Unknown	Kinki	+	*prn*	1	27
BP356	2010	2 mo	0	Kinki	+	*prn*	ND	221
BP362	2010	3 y	Unknown	Kyusyu	+	*prn*	2	27
BP364	2010	8 y	Unknown	Kanto	+	*prn*	2	27
BP367	2010	9 y	Unknown	Kyusyu	+	*prn*	ND	27
BP368	2010	5 mo	Unknown	Kyusyu	+	*prn*	1	186
BP369	2010	15 y	Unknown	Kyusyu	+	*prn*	2	27
BP371	2010	1 mo	0	Kyusyu	+	*prn*	1	186
BP462	2010	6 mo	Unknown	Kanto	+	*prn*	2	27
BP470	2010	1 mo	0	Kanto	+	*prn*	2	27
BP376	2011	2 mo	0	Chubu	+	*prn*	2	27
BP377	2011	1 mo	0	Chugoku	+	*prn*	2	27
BP380	2011	11 y	Unknown	Kyusyu	+	*prn*	2	27
BP388	2011	3 y	Unknown	Kyusyu	+	*prn*	ND	27
BP389	2011	3 mo	1	Kanto	+	*prn*	2	27

Isolate	Isolation year	Patient age	Vaccine status	Origin (district)	Pertactin production	*prn* gene	*prn* allele	MLVA type
BP391	2011	5 y	Unknown	Kanto	+	*prn*	2	27
BP392	2011	4 mo	Unknown	Kanto	+	*prn*	ND	27
BP393	2011	11 mo	Unknown	Kyusyu	+	*prn*	2	27
BP396	2011	1 mo	0	Kinki	+	*prn*	1	187
BP399	2011	2 y	Unknown	Kinki	+	*prn*	ND	27
BP402	2011	1 mo	0	Kinki	+	*prn*	ND	27
BP404	2011	10 y	Unknown	Kyusyu	+	*prn*	2	27
BP405	2011	14 y	Unknown	Kyusyu	+	*prn*	ND	27
BP406	2011	1 mo	0	Kyusyu	+	*prn*	ND	27
BP407	2011	6 mo	Unknown	Kyusyu	+	*prn*	2	27
BP430	2011	1 y	3	Kinki	+	*prn*	2	27
BP431	2011	2 mo	0	Kinki	+	*prn*	9	27
BP432	2011	8 y	0	Kinki	+	*prn*	2	27
BP433	2011	5 mo	0	Kinki	+	*prn*	2	27
BP436	2011	4 mo	0	Kinki	+	*prn*	ND	27
BP453	2011	6 mo	0	Kinki	+	*prn*	1	186
BP463	2011	3 mo	1	Kanto	+	*prn*	2	27
BP471	2011	2 mo	0	Kanto	+	*prn*	2	27
BP493	2011	Unknown	Unknown	Kanto	+	*prn*	ND	27
BP494	2011	0 y	Unknown	Kanto	+	*prn*	ND	27
BP501	2011	Unknown	Unknown	Kinki	+	*prn*	ND	27
BP408	2012	5 mo	Unknown	Kanto	+	*prn*	ND	27
BP409	2012	4 mo	Unknown	Tohoku	+	*prn*	2	27
BP411	2012	17 y	Unknown	Tohoku	+	*prn*	ND	27
BP413	2012	10 y	4	Kanto	+	*prn*	2	27
BP414	2012	1 mo	0	Chubu	+	*prn*	2	27
BP415	2012	8 y	Unknown	Kanto	+	*prn*	ND	27
BP420	2012	4 mo	0	Kanto	+	*prn*	2	27
BP421	2012	5 mo	Unknown	Kanto	+	*prn*	ND	27
BP422	2012	8 y	Unknown	Kanto	+	*prn*	ND	27
BP423	2012	8 y	Unknown	Kanto	+	*prn*	2	27
BP424	2012	9 y	Unknown	Kanto	+	*prn*	ND	27
BP425	2012	7 y	Unknown	Kanto	+	*prn*	ND	27
BP426	2012	8 y	Unknown	Kanto	+	*prn*	ND	27
BP427	2012	4 y	Unknown	Kanto	+	*prn*	ND	27
BP428	2012	10 y	Unknown	Kanto	+	*prn*	ND	27
BP429	2012	12 y	Unknown	Kanto	+	*prn*	ND	27
BP434	2012	7 y	Unknown	Kanto	+	*prn*	2	27
BP435	2012	7 y	Unknown	Kanto	+	*prn*	ND	27
BP439	2012	3 mo	Unknown	Kinki	+	*prn*	2	27
BP441	2012	3 mo	Unknown	Kinki	+	*prn*	ND	186
BP443	2012	3 mo	Unknown	Kinki	+	*prn*	2	27
BP444	2012	1 mo	0	Tohoku	+	*prn*	3	27
BP445	2012	1 mo	0	Tohoku	+	*prn*	2	27
BP446	2012	2 mo	0	Tohoku	+	*prn*	2	27
BP448	2012	2 mo	0	Kinki	+	*prn*	2	27
BP449	2012	4 mo	2	Kinki	+	*prn*	2	27
BP450	2012	1 mo	0	Kinki	+	*prn*	2	27
BP454	2012	11 y	Unknown	Tohoku	+	*prn*	9	27
BP455	2012	1 mo	Unknown	Tohoku	+	*prn*	2	27
BP456	2012	0 mo	0	Tohoku	+	*prn*	2	27
BP472	2012	1 mo	0	Kanto	+	*prn*	2	27
BP477	2012	2 mo	0	Kinki	+	*prn*	ND	27
BP495	2012	0 y	Unknown	Tohoku	+	*prn*	ND	27
BP496	2012	Unknown	Unknown	Kanto	+	*prn*	ND	27
BP513	2012	3 mo	0	Kyusyu	+	*prn*	ND	27
BP514	2012	4 mo	≥1	Kyusyu	+	*prn*	ND	27
BP515	2012	5 mo	≥1	Kyusyu	+	*prn*	ND	26
BP538	2012	0 mo	0	Tohoku	+	*prn*	ND	27
BP473	2013	29 y	≥1	Shikoku	+	*prn*	ND	27
BP474	2013	31 y	Unknown	Kyusyu	+	*prn*	ND	186
BP475	2013	8 y	Unknown	Kyusyu	+	*prn*	ND	28
BP479	2013	10 y	0	Kinki	+	*prn*	ND	27
BP482	2013	4 mo	Unknown	Kanto	+	*prn*	ND	27
BP483	2013	52 y	Unknown	Kyusyu	+	*prn*	ND	27
BP497	2013	3 mo	Unknown	Kanto	+	*prn*	ND	27
BP498	2013	5 y	Unknown	Tohoku	+	*prn*	ND	27
BP516	2013	2 mo	0	Kyusyu	+	*prn*	ND	27

Isolate	Isolation year	Patient age	Vaccine status	Origin (district)	Pertactin production	*prn* gene	*prn* allele	MLVA type
BP484	2014	1 mo	0	Kyusyu	+	*prn*	ND	28
BP485	2014	13 y	Unknown	Kyusyu	+	*prn*	ND	27
BP486	2014	1 mo	0	Shikoku	+	*prn*	ND	27
BP487	2014	1 mo	0	Kanto	+	*prn*	ND	27
BP488	2014	1 mo	0	Chugoku	+	*prn*	ND	27
BP489	2014	5 y	Unknown	Kyusyu	+	*prn*	ND	27
BP490	2014	3 mo	0	Chugoku	+	*prn*	ND	27
BP491	2014	8 y	4	Kinki	+	*prn*	ND	27
BP499	2014	3 mo	Unknown	Kinki	+	*prn*	ND	27
BP500	2014	30 y	Unknown	Kyusyu	+	*prn*	ND	27
BP502	2014	1 mo	0	Kanto	+	*prn*	ND	27
BP503	2014	1 mo	0	Kanto	+	*prn*	ND	27
BP505	2014	36 y	Unknown	Kinki	+	*prn*	ND	27
BP506	2014	10 y	Unknown	Hokkaidou	+	*prn*	ND	27
BP517	2014	1 mo	0	Kyusyu	+	*prn*	ND	27
BP518	2014	1 mo	0	Kyusyu	+	*prn*	ND	27
BP519	2014	8 y	Unknown	Kyusyu	+	*prn*	ND	27
BP520	2014	13 y	Unknown	Kyusyu	+	*prn*	ND	27
BP521	2014	6 y	≥1	Kyusyu	+	*prn*	ND	27
BP522	2014	6 y	≥1	Kyusyu	+	*prn*	ND	27
BP523	2014	8 y	≥1	Kyusyu	+	*prn*	ND	27
BP524	2014	4 mo	Unknown	Kyusyu	+	*prn*	ND	27
BP525	2014	1 mo	0	Kyusyu	+	*prn*	ND	27
BP526	2014	2 mo	0	Kyusyu	+	*prn*	ND	27
BP527	2014	2 mo	0	Kyusyu	+	*prn*	ND	29
BP532	2014	4 mo	0	Kyusyu	+	*prn*	ND	27
BP507	2015	3 mo	Unknown	Kinki	+	*prn*	ND	78
BP508	2015	1 mo	0	Chubu	+	*prn*	ND	27
BP509	2015	32 y	Unknown	Kyusyu	+	*prn*	ND	29
BP511	2015	2 mo	0	Kyusyu	+	*prn*	ND	96
BP512	2015	8 y	4	Kinki	+	*prn*	ND	27
BP528	2015	1 mo	0	Kyusyu	+	*prn*	ND	27
BP529	2015	2 mo	0	Kyusyu	+	*prn*	ND	27
BP530	2015	3 mo	0	Kyusyu	+	*prn*	ND	27
BP531	2015	3 mo	Unknown	Kyusyu	+	*prn*	ND	27
BP534	2015	0 mo	0	Kyusyu	+	*prn*	ND	27
BP536	2015	3 mo	1	Shikoku	+	*prn*	ND	27
BP537	2015	2 mo	0	Kinki	+	*prn*	ND	31
BP539	2015	1 mo	0	Tohoku	+	*prn*	ND	32
BP540	2015	1 mo	0	Tohoku	+	*prn*	ND	27
BP541	2015	6 mo	0	Kinki	+	*prn*	ND	27
BP551	2015	12 y	3	Chubu	+	*prn*	2	27
BP542	2016	9 y	4	Hokkaidou	+	*prn*	2	27
BP543	2016	10 y	Unknown	Kinki	+	*prn*	2	27
BP544	2016	1 mo	0	Kyusyu	+	*prn*	1	34
BP545	2016	13 y	4	Chubu	+	*prn*	2	27
BP549	2016	9 y	4	Hokkaidou	+	*prn*	2	27
BP555	2016	6 mo	3	Chubu	+	*prn*	2	28
BP556	2016	8 y	4	Kanto	+	*prn*	2	27

*, negative; +, positive; ND, not determined; MLVA, multilocus variable number tandem repeat analysis.

Technical Appendix Table 2. Primers used in this study of *Bordetella pertussis* isolates, Japan, 2008–2016*

Designation	Primer name	Sequence (5′ to 3′)
Conventional PCR	SS-defect-F5	CTCTGTCACGCATTGACAAC
	SS-outerR	CTCGGCCGCGGGATTTTCTA
	IS*481*-combine-F	GCAGACGCCACTAGGTGTGA
	IS*481*-combine-R	AAAGGTCGCCGCGCTGCCTA
qRT PCR	qprn-F	ATCGTCAAGACCGGTGAGCG
	qprn-R	CTGACGGCCGCTTACCTTGA
	qrecA-F	CCAATGTGGTCGACAAGTCC
	qrecA-R	ATGGCCATTTCCTTGTGCTC

*qRT–PCR, quantitative reverse transcription PCR.

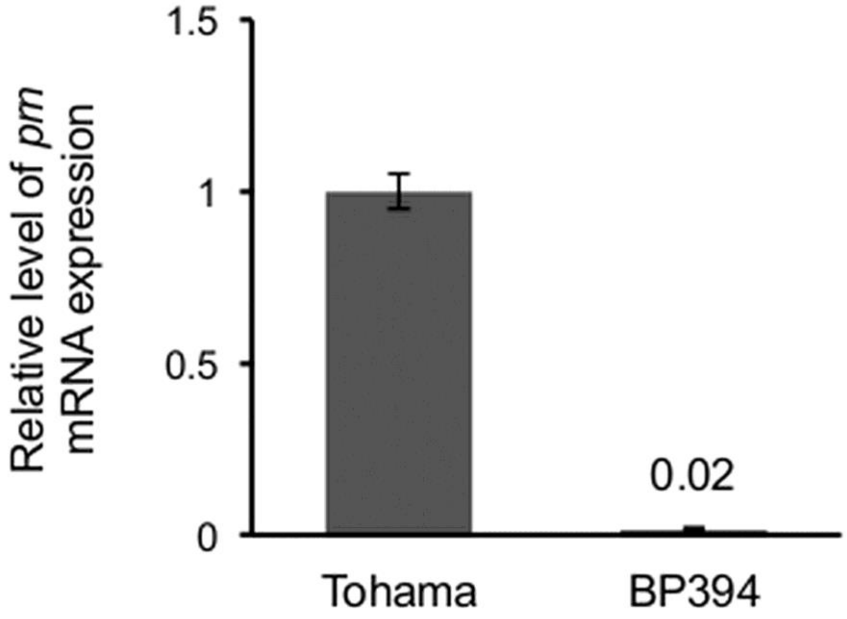

Technical Appendix Figure. Lack of *prn* transcript expression in pertactin–deficient *Bordetella pertussis* isolate BP394. The pertactin–deficient isolate was cultured for 3 days on Bordet-Gengou agar. Total RNA was isolated by using the RNeasy Mini Kit (QIAGEN, Hilden, Germany) and treated with RNase–Free DNase (QIAGEN) to degrade contaminating DNA. Total RNA (0.1 μg) was reverse–transcribed into cDNA by using the PrimeScript RT Master Mix with random hexamers (TaKaRa Bio, Inc., Shiga, Japan). Relative levels of *prn* and *recA* transcripts were determined using SYBR Premix Ex *Taq*II (TaKaRa Bio) with the ABI PRISM 7500 Fast Real–Time PCR System (Applied Biosystems, Waltham, MA, USA). Quantitative reverse transcription–PCR (qRT–PCR) conditions were 10 s at 95°C, followed by 40 cycles of 95°C for 3 s, and 60°C for 30 s. Primer sets qprn–F/qprn–R and qrecA–F/qrecA–R were used for *prn* and *recA* amplification, respectively (Technical Appendix Table 2). The relative *prn* transcript level was calculated using the ΔΔCt method and was normalized to that of *recA*. The *recA* transcript was used as an internal control for each sample. Data are presented as -fold- changes in expression compared with

those observed in *B. pertussis* strain Tohama. The mean ± SDs of results obtained from 3 separate experiments are shown.
